# Impact of *VEGFA* polymorphisms on glioma risk in Chinese

**DOI:** 10.18632/oncotarget.19380

**Published:** 2017-07-19

**Authors:** Peng Zhao, Anjing Chen, Qichao Qi, Wenjing Zhou, Zichao Feng, Jiwei Wang, Ning Yang, Xingang Li, Jian Wang, Qibing Huang, Bin Huang

**Affiliations:** ^1^ Department of Neurosurgery, Qilu Hospital of Shandong University, Jinan 250012, China; ^2^ Brain Science Research Institute, Shandong University, Jinan 250012, China; ^3^ Department of Biomedicine, University of Bergen, Bergen 5009, Norway; ^4^ Department of Emergency Surgery, Qilu Hospital of Shandong University, Jinan 250012, China

**Keywords:** genetic susceptibility, VEGFA, glioma, single nucleotide polymorphism

## Abstract

Several single nucleotide polymorphisms (SNPs) in the vascular endothelial growth factor A (VEGFA) gene have been previously reported to be associated with glioma susceptibility, but individual studies have demonstrated inconclusive results. In the current study, a meta-analysis was performed to derive a more precise estimation of the involvement of *VEGFA* polymorphisms in glioma development. A comprehensive literature search conducted in PubMed, Embase, the Cochrane Library, and OVID databases through February 25, 2017 yielded 4 eligible studies consisting of 2,275 cases and 2,475 controls. Pooled odds ratios (ORs) and corresponding 95% confidence intervals (CIs) were calculated under allele contrast, dominant, recessive, homozygous, and heterozygous models. In general, minor alleles of polymorphisms *rs3025039*, *rs2010963*, and *rs3025030* were associated with increased glioma risk. In contrast, a significant correlation was found between the minor allele of polymorphism *rs3024994* and decreased susceptibility to glioma. Moreover, statistically significant associations with glioma risk were observed for polymorphisms *rs1413711* and *rs3025035* in the meta-analysis although positive associations were not observed in any of the included studies individually. No significant correlations with glioma susceptibility were identified for polymorphisms *rs3025010* or *rs833069* except in the recessive model. Finally, stratified analysis on the basis of genotyping method and Hardy-Weinberg equilibrium (HWE) in controls revealed no significant difference between subgroups. Our results indicated that several VEGFA polymorphisms might be risk factors for glioma in Chinese. More studies with larger sample sizes using different ethnicities are needed to provide additional evidence.

## INTRODUCTION

Glioma is the most common type of primary central nervous system tumors and has an invariable fatal outcome and dismal prognosis [1, 2]. Despite optimal treatment, the median survival is only 12–15 months for patients with glioblastomas and 2–5 years for patients with anaplastic gliomas [3, 4].

The etiology of glioma is still poorly understood. To date, exposure to ionizing radiation is the only clearly known environmental risk factor, which accounts for only a few cases [5]. In recent years, genome-wide association studies (GWASs) have been carried out to examine the genetic component of susceptibility to glioma. Significant associations were observed between glioma risk and single nucleotide polymorphisms (SNPs) in several genes, including telomerase reverse transcriptase (TERT), regulator of telomerase elongation helicase 1 (RTEL 1), cyclin dependent kinase inhibitor 2A-cyclin dependent kinase inhibitor 2B (CDKN2A-CDKN2B), coiled-coil domain containing 26 (CCDC26), tumor protein p53 (TP53), epidermal growth factor receptor (EGFR), and pleckstrin homology-like domain family B member 1 (PHLDB1) [6–8]. However, additional factors that contribute to glioma susceptibility require further investigation.

Angiogenesis is a critical process in the development of human glioma, which promotes survival, malignancy, and growth of tumor cells. The vascular endothelial growth factor A (VEGFA) gene, which is located on chromosome 6p12 and consists of 9 exons, is one of the most predominant mediators of pathologic angiogenesis. First, endothelial cells are stimulated by VEGF to proliferate and to migrate [9]. Second, expression of anti-apoptotic factors such as B cell CLL/lymphoma 2 (Bcl-2) and survivin is induced by VEGFA [10]. Third, VEGFA has been shown to increase vascular permeability allowing plasma proteins and other circulating macromolecules to cross the endothelium [11, 12]. Finally, numerous studies have demonstrated that VEGFA is also a possible mediator of tumor-induced angiogenesis in glioma [13, 14]. Based on these roles in glioma development, over 30 SNPs in the *VEGFA* gene have been described. Recent studies have examined the associations of specific SNPs in the *VEGFA* gene with glioma risk, but the significance of these findings remains unclear.

Due to insufficient population sizes, the statistical power of each study was relatively low, and evidence of the risk associated with each polymorphism was inconclusive. To increase statistical power, we conducted a systematic review and meta-analysis of published studies investigating the associations between *VEGFA* polymorphisms and glioma susceptibility.

## RESULTS

### Study characteristics

The search strategy identified 104 studies, but only 9 full-text articles were chosen for further detailed evaluation [15–23]. Five of these studies were excluded, including two that did not investigate the polymorphism of interest [18, 20], one that did not provide exact genotypes [15], and two that investigated the association between VEGFA polymorphisms and the prognosis of glioma [21, 22]. Finally, four studies [16, 17, 19, 23], including 2275 cases and 2475 controls, were included for analysis. The following is the breakdown of the number of studies and individuals that met our eligibility criteria for each polymorphism evaluated: *rs3025039*, 4 case-control studies with 2275 cases and 2475 controls; *rs2010963*, 3 case-control studies with 2115 cases and 2155 controls; *rs3024994*, 2 case-control studies with 1235 cases and 1275 controls; *rs1413711*, 2 case-control studies with 1235 cases and 1275 controls; *rs833069*, 2 case-control studies with 1235 cases and 1275 controls; *rs3025010*, 2 case-control studies with 1235 cases and 1275 controls; *rs3025030*, 2 case-control studies with 1235 cases and 1275 controls; and *rs3025035*, 2 case-control studies with 1235 cases and 1275 controls (Table 1). All studies scored a value ≥ 7 (high-quality) as determined in the Newcastle-Ottawa Scale, and all individuals were of Chinese descent.

**Table 1:** Characteristics of individual studies evaluating association between *VEGFA* polymorphisms and glioma risk

Single Nucleotide Polymorphisms	Author	Year	Ethnicity	Source of Controls	Genotyping Method	Quality Score	Cases	Controls	*P* for HWE in controls	Cases			Controls		
										AA	AB	BB	AA	AB	BB
rs3025039	Zhang	2015	Chinese	HB	MALDI-TOF mass spectrometry	8	477	477	0.668	329	128	20	360	110	7
rs3025039	Jiang	2013	Chinese	HB	PCR-RFLP	7	880	880	< 0.001	550	242	88	572	255	53
rs3025039	Li	2011	Chinese	HB	MALDI-TOF mass spectrometry	8	758	798	0.220	529	202	27	563	220	15
rs3025039	Bao	2011	Chinese	HB	PCR-RFLP	8	160	320	0.396	113	38	9	231	84	5
rs2010963	Zhang	2015	Chinese	HB	MALDI-TOF mass spectrometry	8	477	477	0.470	155	240	82	185	230	62
rs2010963	Jiang	2013	Chinese	HB	PCR-RFLP	7	880	880	< 0.001	448	257	175	485	255	140
rs2010963	Li	2011	Chinese	HB	MALDI-TOF mass spectrometry	8	758	798	0.893	247	393	120	306	379	115
rs3024994	Zhang	2015	Chinese	HB	MALDI-TOF mass spectrometry	8	477	477	0.073	438	34	5	423	50	4
rs3024994	Li	2011	Chinese	HB	MALDI-TOF mass spectrometry	8	758	798	0.370	691	54	2	717	84	4
rs1413711	Zhang	2015	Chinese	HB	MALDI-TOF mass spectrometry	8	477	477	0.056	229	180	68	244	182	51
rs1413711	Li	2011	Chinese	HB	MALDI-TOF mass spectrometry	8	758	798	0.468	411	286	61	452	306	45
rs833069	Zhang	2015	Chinese	HB	MALDI-TOF mass spectrometry	8	477	477	0.892	100	261	116	118	237	122
rs833069	Li	2011	Chinese	HB	MALDI-TOF mass spectrometry	8	758	798	0.499	126	411	214	129	393	271
rs3025010	Zhang	2015	Chinese	HB	MALDI-TOF mass spectrometry	8	477	477	0.258	233	193	51	246	186	45
rs3025010	Li	2011	Chinese	HB	MALDI-TOF mass spectrometry	8	758	798	0.820	382	307	71	436	317	60
rs3025030	Zhang	2015	Chinese	HB	MALDI-TOF mass spectrometry	8	477	477	0.108	319	140	18	317	150	10
rs3025030	Li	2011	Chinese	HB	MALDI-TOF mass spectrometry	8	758	798	0.055	522	204	30	551	240	15
rs3025035	Zhang	2015	Chinese	HB	MALDI-TOF mass spectrometry	8	477	477	0.760	356	114	7	332	133	12
rs3025035	Li	2011	Chinese	HB	MALDI-TOF mass spectrometry	8	758	798	0.123	559	191	8	575	226	14

HB, hospital-based; HWE, Hardy-Weinberg Equilibrium; MALDI-TOF, matrix assisted laser desorption ionization time-of-flight; PCR-RFLP, polymerase chain reaction-restriction fragment length polymorphism.

### Quantitative synthesis

Heterogeneity among studies, as measured by the *Q* test and the *I*^2^ statistic, was not significant for any *VEGFA* polymorphism (Table 2). Therefore, the fixed-effect model and the Mantel-Haenszel method were used to calculate the pooled OR.

**Table 2:** ORs and 95% CI for association of *VEGFA* polymorphisms with glioma susceptibility under different genetic models

Genetic models	*N*	OR [95% CI]	*P* (OR)	Model (method)	*I*-square (%)	*P* (H)	*P* (Begg)	*P* (Egger)
*rs3025039*								
Allele contrast	4	**1.209 [1.088–1.343]**	**< 0.001**	F (M-H)	0.0	0.425	0.734	0.600
Dominant model	4	1.131 [1.000–1.280]	0.050	F (M-H)	0.0	0.455	1.000	0.713
Recessive model	4	**1.973 [1.489–2.615]**	**< 0.001**	F (M-H)	0.0	0.456	0.089	0.047
Homozygous model	4	**1.982 [1.491–2.635]**	**< 0.001**	F (M-H)	0.0	0.424	0.089	0.054
Heterozygous model	4	1.028 [0.902–1.171]	0.677	F (M-H)	0.0	0.463	1.000	0.838
*rs2010963*								
Allele contrast	3	**1.197 [1.096–1.308]**	**< 0.001**	F (M-H)	0.0	0.837	1.000	0.450
Dominant model	3	**1.247 [1.102–1.411]**	**< 0.001**	F (M-H)	0.0	0.764	0.296	0.382
Recessive model	3	**1.256 [1.067–1.479]**	**0.006**	F (M-H)	0.0	0.572	1.000	0.818
Homozygous model	3	**1.375 [1.153–1.639]**	**< 0.001**	F (M-H)	0.0	0.724	1.000	0.458
Heterozygous model	3	**1.197 [1.046–1.369]**	**0.009**	F (M-H)	0.0	0.553	1.000	0.774
*rs3024994*								
Allele contrast	2	**0.698 [0.539–0.904]**	**0.006**	F (M-H)	0.0	0.670	1.000	-
Dominant model	2	**0.675 [0.514–0.886]**	**0.005**	F (M-H)	0.0	0.847	1.000	-
Recessive model	2	0.901 [0.325–2.497]	0.840	F (M-H)	0.0	0.441	1.000	-
Homozygous model	2	0.868 [0.313–2.409]	0.786	F (M-H)	0.0	0.442	1.000	-
Heterozygous model	2	**0.663 [0.501–0.878]**	**0.004**	F (M-H)	0.0	0.958	1.000	-
*rs1413711*								
Allele contrast	2	**1.143 [1.010–1.293]**	**0.034**	F (M-H)	0.0	0.768	1.000	-
Dominant model	2	1.105 [0.944- 1.293]	0.213	F (M-H)	0.0	0.798	1.000	-
Recessive model	2	**1.430 [1.083–1.888]**	**0.012**	F (M-H)	0.0	0.833	1.000	-
Homozygous model	2	**1.455 [1.092–1.940]**	**0.011**	F (M-H)	0.0	0.870	1.000	-
Heterozygous model	2	1.037 [0.878–1.225]	0.666	F (M-H)	0.0	0.887	1.000	-
*rs833069*								
Allele contrast	2	0.944 [0.844–1.055]	0.308	F (M-H)	56.3	0.131	1.000	-
Dominant model	2	1.077 [0.881–1.316]	0.471	F (M-H)	32.4	0.224	1.000	-
Recessive model	2	**0.823 [0.691–0.979]**	**0.028**	F (M-H)	11.2	0.288	1.000	-
Homozygous model	2	0.924 [0.731–1.167]	0.505	F (M-H)	44.7	0.179	1.000	-
Heterozygous model	2	1.166 [0.944–1.439]	0.155	F (M-H)	0.0	0.373	1.000	-
*rs3025010*								
Allele contrast	2	1.125 [0.996–1.270]	0.059	F (M-H)	0.0	0.774	1.000	-
Dominant model	2	1.133 [0.969–1.325]	0.116	F (M-H)	0.0	0.875	1.000	-
Recessive model	2	1.231 [0.936–1.618]	0.137	F (M-H)	0.0	0.677	1.000	-
Homozygous model	2	1.284 [0.968–1.704]	0.083	F (M-H)	0.0	0.679	1.000	-
Heterozygous model	2	1.102 [0.934–1.299]	0.250	F (M-H)	0.0	0.959	1.000	-
*rs3025030*								
Allele contrast	2	1.048 [0.906–1.212]	0.526	F (M-H)	0.0	0.959	1.000	-
Dominant model	2	0.974 [0.823–1.151]	0.754	F (M-H)	0.0	0.941	1.000	-
Recessive model	2	**2.037 [1.248–3.324]**	**0.004**	F (M-H)	0.0	0.734	1.000	-
Homozygous model	2	**1.980 [1.210–3.240]**	**0.007**	F (M-H)	0.0	0.748	1.000	-
Heterozygous model	2	0.909 [0.764–1.081]	0.280	F (M-H)	0.0	0.855	1.000	-
*rs3025035*								
Allele contrast	2	**0.829 [0.709–0.969]**	**0.019**	F (M-H)	0.0	0.603	1.000	-
Dominant model	2	**0.824 [0.692–0.981]**	**0.030**	F (M-H)	0.0	0.618	1.000	-
Recessive model	2	0.595 [0.313–1.128]	0.112	F (M-H)	0.0	0.932	1.000	-
Homozygous model	2	0.567 [0.298–1.078]	0.084	F (M-H)	0.0	0.906	1.000	-
Heterozygous model	2	0.843 [0.705–1.007]	0.059	F (M-H)	0.0	0.655	1.000	-

OR, odds ratio; CI, confidence intervals; *N*, number of included studies; F, fixed-effect model; M-H, Mantel-Haenszel method; *P* (H), *P* for heterogeneity. *P* values < 0.05 were considered as statistically significant, and are highlighted in bold font in the table.

**Figure 1:**
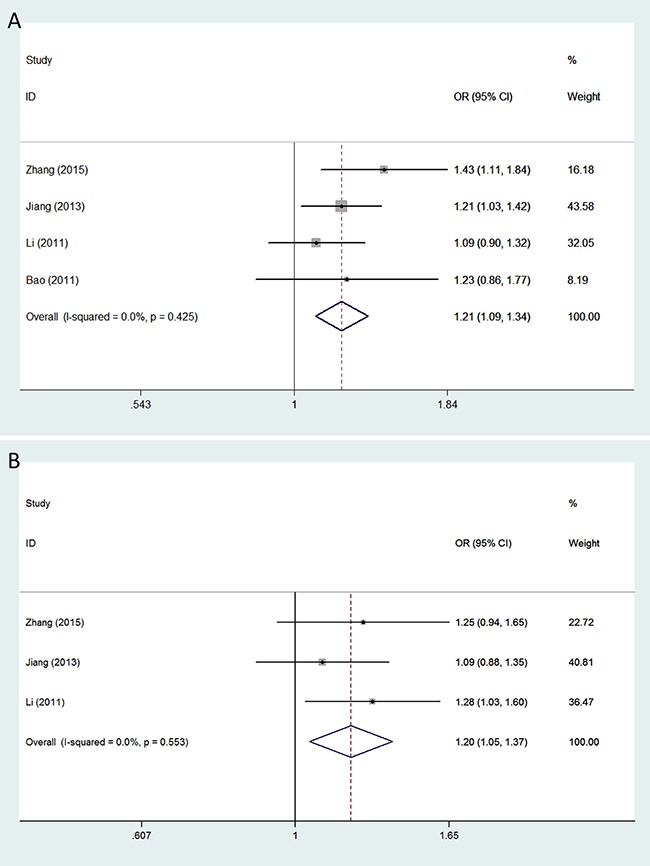
Forest plots of glioma risk associated with *VEGFA* polymorphisms *rs3025039* and *rs2010963* Models represented include (**A**) *rs3025039* (allele contrast model) and (**B**) *rs2010963* (heterozygous model). The area of the squares reflects the weight (inverse of the variance). The diamond represents the summary OR and 95% CI.

**Figure 2:**
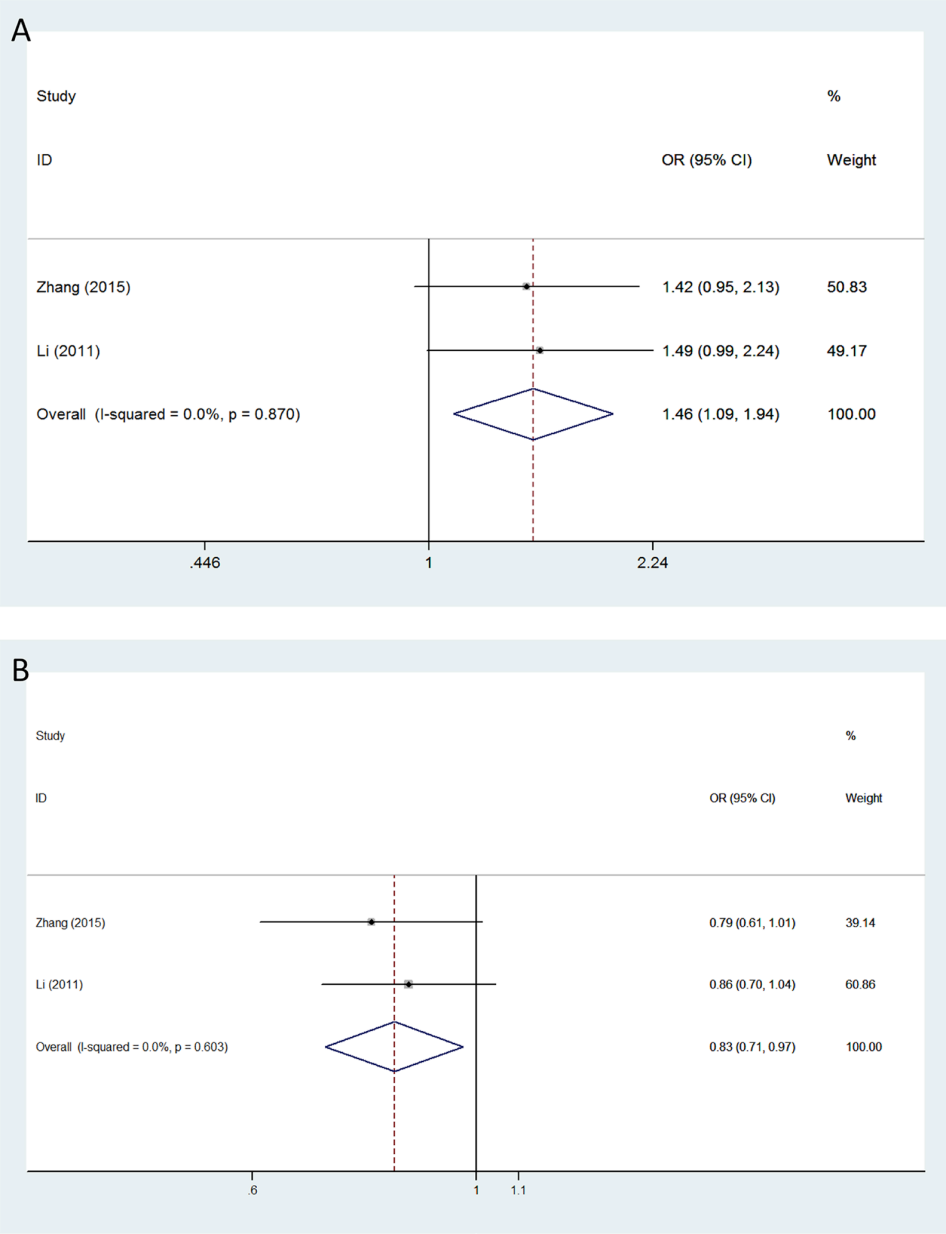
Forest plots of glioma risk in different genetic models associated with *VEGFA* polymorphisms *rs1413711* and *rs3025035* Models represented include (**A**) *rs1413711* (homozygous model) and (**B**) *rs3025035* (allele contrast model). The area of the squares reflects the weight (inverse of the variance). The diamond represents the summary OR and 95% CI.

The minor allele of *VEGFA rs3025039* was related to a significantly increased glioma risk under the allele contrast (OR = 1.209, 95% CI = 1.088–1.343, *P* < 0.001), recessive (OR = 1.973, 95% CI = 1.489–2.615, *P* < 0.001), and homozygous models (OR = 1.982, 95% CI = 1.491–2.635, *P* < 0.001) (Figure 1A). A significant association between polymorphism *rs2010963* and glioma susceptibility was observed under all genetic models: allele contrast (OR = 1.197, 95% CI = 1.096–1.308, *P* < 0.001); dominant (OR = 1.247, 95% CI = 1.102–1.411, *P* < 0.001); recessive (OR = 1.256, 95% CI = 1.067–1.479, *P* = 0.006); homozygous (OR = 1.375, 95% CI = 1.153–1.639, *P* < 0.001); and heterozygous (OR = 1.197, 95% CI = 1.046–1.369, *P* = 0.009) (Figure 1B). The minor allele of polymorphism *rs3024994* was associated with decreased glioma risk under the allele contrast (OR = 0.698, 95% CI = 0.539–0.904, *P* = 0.006), dominant (OR = 0.675, 95% CI = 0.514–0.886, *P* = 0.005), and homozygous models (OR = 0.663, 95% CI = 0.501–0.878, *P* = 0.004). Polymorphism *rs3025030* was significantly associated with glioma susceptibility in the recessive (OR = 2.037, 95% CI = 1.248–3.324, *P* = 0.004) and homozygous models (OR = 1.980, 95% CI = 1.210–3.240, *P* = 0.007). Moreover, significant impacts of polymorphisms *rs1413711* and *rs3025035* on glioma susceptibility were observed although neither of the included studies showed any positive associations (Figure 2): for *rs1413711*, allele contrast (OR = 1.143, 95% CI = 1.010–1.293, *P* = 0.034), recessive (OR = 1.430, 95% CI = 1.083–1.888, *P* = 0.012), and homozygous models (OR = 1.455, 95% CI = 1.092–1.940, *P* = 0.011); for *rs3025035*, allele contrast (OR = 0.829, 95% CI = 0.709–0.969, *P* = 0.019) and dominant models (OR = 0.824, 95% CI = 0.692–0.981, *P* = 0.030). However, no significant associations were identified for polymorphisms *rs3025010* or *rs833069*, except in the recessive model, and glioma susceptibility (Table 2).

### Subgroup analysis

Because of the limited number of eligible studies, stratified analysis on the basis of genotyping method and Hardy-Weinberg equilibrium (HWE) in controls was only performed on *rs3025039*. A significant association between *VEGFA* polymorphism *rs3025039* and glioma susceptibility was identified in HWE group under the allele contrast (OR = 1.211, 95% CI = 1.053–1.392, *P* = 0.007), recessive (OR = 2.451, 95% CI = 1.537–3.907, *P* < 0.001), and homozygous models (OR = 2.482, 95% CI = 1.554–3.964, *P* < 0.001) (Table 3).

**Table 3:** Subgroup analyses for association of *VEGFA rs3025039* with glioma susceptibility under different genetic models

Subgroups	*N*	OR [95% CI]	*P* (OR)	Model (method)	*I*-square (%)	*P* (H)	*P* (Begg)	*P* (Egger)
Allele contrast								
Overall	4	**1.209 [1.088–1.343]**	**< 0.001**	F (M-H)	0.0	0.425	0.734	0.600
MALDI-TOF mass spectrometry	2	**1.207 [1.037–1.404]**	**0.015**	F (M-H)	64.0	0.096	-	-
PCR-RFLP	2	**1.211 [1.047–1.401]**	**0.010**	F (M-H)	0.0	0.919	-	-
HWE	3	**1.211 [1.053–1.392]**	**0.007**	F (M-H)	28.3	0.248	-	-
Dominant model								
Overall	4	1.131 [1.000–1.280]	0.050	F (M-H)	0.0	0.455	1.000	0.713
MALDI-TOF mass spectrometry	2	1.154 [0.971–1.371]	0.105	F (M-H)	59.9	0.114	-	-
PCR-RFLP	2	1.108 [0.929–1.322]	0.254	F (M-H)	0.0	0.893	-	-
HWE	3	1.143 [0.974–1.340]	0.101	F (M-H)	22.3	0.276	-	-
Recessive model								
Overall	4	**1.973 [1.489–2.615]**	**< 0.001**	F (M-H)	0.0	0.456	0.089	0.047
MALDI-TOF mass spectrometry	2	**2.254 [1.350–3.764]**	**0.002**	F (M-H)	0.0	0.444	-	-
PCR-RFLP	2	**1.859 [1.327–2.605]**	**< 0.001**	F (M-H)	40.8	0.194	-	-
HWE	3	**2.451 [1.537–3.907]**	**< 0.001**	F (M-H)	0.0	0.528	-	-
Homozygous model								
Overall	4	**1.982 [1.491–2.635]**	**< 0.001**	F (M-H)	0.0	0.424	0.089	0.054
MALDI-TOF mass spectrometry	2	**2.297 [1.373–3.843]**	**0.002**	F (M-H)	0.0	0.376	-	-
PCR-RFLP	2	**1.852 [1.315–2.608]**	**< 0.001**	F (M-H)	37.4	0.206	-	-
HWE	3	**2.482 [1.554–3.964]**	**< 0.001**	F (M-H)	0.0	0.504	-	-
Heterozygous model								
Overall	4	1.028 [0.902–1.171]	0.677	F (M-H)	0.0	0.463	1.000	0.838
MALDI-TOF mass spectrometry	2	1.077 [0.901–1.288]	0.416	F (M-H)	48.8	0.162	-	-
PCR-RFLP	2	0.975 [0.806–1.180]	0.796	F (M-H)	0.0	0.795	-	-
HWE	3	1.054 [0.893–1.244]	0.532	F (M-H)	14.5	0.310	-	-

OR, odds ratio; CI, confidence intervals; *N*, number of included studies; F, fixed-effect model; M-H, Mantel-Haenszel method; *P* (H), *P* for heterogeneity; MALDI-TOF, matrix assisted laser desorption ionization time-of-flight; PCR-RFLP, polymerase chain reaction-restriction fragment length polymorphism. *P* values < 0.05 were considered as statistically significant and are highlighted in bold font in the table.

In the subgroup analysis based on the genotyping method, positive correlations were observed between the *rs3025039* polymorphism and glioma susceptibility in both matrix assisted laser desorption ionization time-of-flight (MALDI-TOF) mass spectrometry and polymerase chain reaction-restriction fragment length polymorphism (PCR-RFLP) groups (Table 3).

### Sensitivity analysis

To further validate the robustness of the outcomes, we conducted sensitivity analyses by repeating the pooled analysis while sequentially omitting each study included individually for all genetic models. Sequential removal of single studies did not result in significant changes in the combined ORs, suggesting that the results of this meta-analysis were stable and robust (data not shown).

### Publication bias

**Figure 3:**
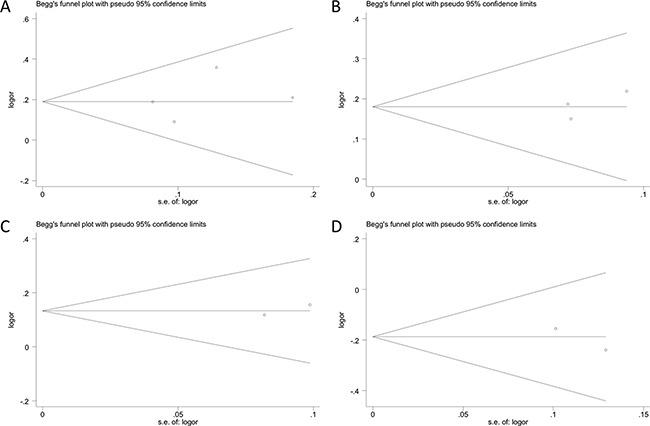
Begg's funnel plots assessing evidence of publication bias from the eligible studies Polymorphisms represented include (**A**) *rs3025039*, (**B**) *rs2010963*, (**C**) *rs1413711*, and (**D**) *rs3025035* in allele contrast model. Each circle represents an individual study for the indicated association. No publication bias was observed.

No obvious asymmetry was observed in any of the Begg's funnel plots, which indicates that publication bias was generally not influencing the results (Figure 3). All *P* values from the Egger's and the Begg's tests are listed in Table 2. The values are consistent with the absence of significant publication bias in the analysis of glioma risk and polymorphisms in most genotype models, except for *rs3025039* in the recessive model. However, analysis with the trim and fill method demonstrated that the results of our study did not significantly change after adjusting for publication bias.

## DISCUSSION

In the present study, we assessed the contribution of 8 *VEGFA* SNPs to the risk of glioma and observed several positive associations at 6 SNPs. The minor alleles of polymorphisms *rs3025039*, *rs2010963*, and *rs3025030* were associated with increased glioma risk. In contrast, a significant correlation was found between the minor allele of polymorphism *rs3024994* and decreased susceptibility to glioma. Finally, polymorphisms *rs1413711* and *rs3025035* significantly influenced glioma risk, although no positive associations were observed for any of the included studies. These results demonstrate that *VEGFA* polymorphisms may be associated with glioma risk.

Tumor cells require angiogenesis to deliver nutrients and oxygen to support their rapid growth and high metabolism. VEGFA is an important pro-angiogenic factor for tumor progression because it promotes endothelial cell proliferation and remodels the extracellular matrix in blood vessels [24]. Elevated VEGFA expression is correlated with increased tumor microvessel density [25], high tumor grade, and poor prognosis in gliomas [26–28]. Of particular interest were polymorphisms located in regions of the gene that might possibly affect VEGFA RNA and/or protein levels. The polymorphisms *rs2010963* and *rs3025039* are located in the 5′-untranslated (5’-UTR) and 3’-untranslated (3′-UTR) regions of the *VEGFA* gene, respectively. Renner *et al*. first identified the *VEGFA rs3025039* polymorphism and found that the T allele led to significantly lower plasma VEGFA levels in healthy young men [29]. A functional study revealed that variants of *rs2010963* led to a higher production of the VEGFA precursor and VEGFA in several cell lines [30]. Such changes in VEGFA expression levels might contribute to glioma development. However, the exact mechanisms by which these polymorphisms affect glioma risk remains unclear.

The genetic variants within conventional regulatory regions, such as the 5′- and 3′-UTRs, have been the focus in most previous studies evaluating risk associations; however, accumulating evidence highlights the importance of intronic *VEGFA* polymorphisms as markers of disease susceptibility [31, 32]. Variants of *rs3024994* and *rs3025030* polymorphisms, which are located in intronic regions, were reported to lead to changes in potential binding sites of transcription factors. Such base pair changes may result in aberrant VEGFA expression and ultimately tumorigenesis [17].

No individual eligible studies focused on *VEGFA* polymorphisms *rs1413711* and *rs3025035* revealed any statistically significant positive correlations associated with glioma risk. Yet pooled analysis demonstrated that both polymorphisms might be related to glioma risk in Chinese. This discrepancy may result from the limited sample size of individual studies. Pooling of cases through meta-analysis however potentially reveals positive associations by narrowing the 95% CI. The polymorphisms *rs1413711* and *rs3025035* are also located in intronic regions of the *VEGFA* gene; however, no functional assay has been performed to investigate whether these polymorphisms influence VEGFA expression or function to date. Therefore, larger, preferably population-based case-control studies, as well as mechanistic studies involving variants of VEGFA on glioma progression, are warranted to validate our findings and to further investigate their roles in the development of glioma.

A sufficient number of cases and controls were pooled from different studies and a more accurate estimation of the associations between *VEGFA* SNPs and glioma risk compared to individual studies was evaluated. Moreover, we also revealed some positive associations that were not observed in previous work. However, the present study also has several limitations that should be taken into account when interpreting the findings. First, our analysis was limited to articles published in English and individuals of Chinese descent. Language bias may therefore exist, and it remains unclear whether these results can be generalized to other populations. Second, significant publication bias was observed in the analysis of glioma risk and *VEGFA rs3025039* in the recessive model. The trim and fill method was carried out to adjust the pooled results. Finally, although subgroup analysis was performed based on genotyping method and HWE in controls, we did not design further analyses to investigate associations between *VEGFA* SNPs and glioma subtype because of limited data.

In conclusion, the statistical data presented in this study suggest that variants of the *VEGFA* gene might be important in promoting the development of glioma in Chinese. Subsequent studies with larger sample sizes using different ethnicities are needed to evaluate the association between *VEGFA* gene polymorphisms and various types of glioma.

## MATERIALS AND METHODS

### Search strategy

Data analyzed in this meta-analysis were provided by multiple case-control studies systematically identified by searching PubMed, Embase, the Cochrane Library, and OVID databases up to February 25, 2017. The standardized search strategy included the use of MeSH terms (“Vascular Endothelial Growth Factor A”, “Glioma”, and “Polymorphism, Single Nucleotide”) or key words related to polymorphism (polymorphism or polymorphisms or variation or variations or variant or variants or mutation or mutations or genotype or genotypes), the disease (glioma or gliomas or glioblastoma or glioblastomas or astrocytoma or astrocytomas or oligodendroglioma or oligodendrogliomas), or the gene (VEGF or vascular endothelial growth factor).

### Inclusion criteria

Inclusion criteria for studies were the following: (1) a case-control or cohort study, (2) contain sufficient data for the evaluation of VEGF polymorphism on glioma risk, (3) published in English, and (4) performed in humans.

### Data extraction

Two reviewers (P. Z. and A. C.) independently selected studies and extracted the following data from each study: first author's surname, publication year, ethnicity, glioma types, numbers of cases and controls, and the genotype distributions of cases and controls. Any disagreements were resolved by discussion with a third investigator (Q. Q.).

### Quality assessment

According to the Newcastle-Ottawa Scale and Agency for Healthcare Research and Quality (http://www.ohri.ca/programs/clinical_epidemiology/oxford.asp), the quality of the eligible studies was separately evaluated by two investigators (P. Z. and A. C.). Any disagreements were resolved by consensus through discussion.

### Statistical analysis

We used STATA software 11.0 (STATA Corp., College Station, TX, USA) for all statistical analyses. The pooled odds ratios (OR) and 95% confidence intervals (CI) were calculated to evaluate the strength of associations between *VEGFA* polymorphisms and glioma risk under allele contrast (B *vs* A), dominant (BB + AB *vs* AA), recessive (BB *vs* AB + AA), homozygous (BB *vs* AA), and heterozygous models (AB *vs* AA). Between-study heterogeneity was assessed by the *I*^2^ statistic and *Q* test with *P* < 0.05 and *I*^2^ > 50% indicating evidence of heterogeneity. The fixed-effect (Mantel-Haenszel method) or random-effects model (DerSimonian-Laird method) was used to calculate the pooled effect estimates in the presence or absence of heterogeneity. Sensitivity analysis was conducted by sequentially excluding each study to assess the stability of the results. The Begg's and the Egger's tests were performed to assess publication bias. All tests were two-tailed, and *P*-values < 0.05 were considered statistically significant.
